# Research advances in hydrogel-based strategies for thyroid disease management: from diagnosis to therapeutic applications

**DOI:** 10.3389/fbioe.2026.1735751

**Published:** 2026-01-26

**Authors:** Lei Tang, Jing Shen

**Affiliations:** 1 Department of Thyroid Surgery, The Second Affiliated Hospital of Jiaxing University, Jiaxing, China; 2 Department of Breast Surgery, The Second Affiliated Hospital of Jiaxing University, Jiaxing, China

**Keywords:** diagnosis, drug delivery, hydrogel, management, thyroid disease, tissue engineering, treatment

## Abstract

This comprehensive review examines cutting-edge developments in hydrogel technology for thyroid disease management, encompassing both diagnostic and therapeutic applications. As a promising polymeric biomaterial with a three-dimensional (3D) network structure, hydrogels demonstrate exceptional potential in thyroid disease due to their unique combination of properties: (1) remarkable biocompatibility, (2) precisely tunable physicochemical characteristics, and (3) controlled drug release capabilities. Our analysis systematically evaluates hydrogel applications across the spectrum of thyroid disorders, including (i) diagnostic approaches for thyroid nodules, (ii) therapeutic interventions for endocrine dysfunction (hyperthyroidism, hypothyroidism, and post-thyroidectomy hypoparathyroidism), (iii) innovative treatments for thyroid neoplasms, and (iv) hemostasis, wound healing, repair of thyroid cartilage and laryngeal nerve injuries following thyroid surgery. It focuses on analyzing their advantages and challenges in drug delivery, minimally invasive therapy, tissue engineering, and postoperative care. Finally, future development directions for hydrogels in the field of thyroid disease are discussed.

## 1 Introduction

**FIGURE 1:**
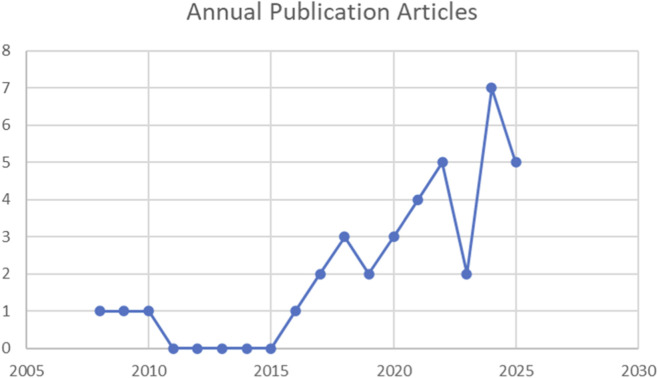
Annual publication volume of hydrogel applications in the thyroid field (2008–2025).

Thyroid disease is a prevalent endocrine disorder affecting individuals across all age groups, genders, and ethnicities, with a broad spectrum of manifestations including hyperthyroidism, hypothyroidism, thyroiditis, and benign or malignant thyroid neoplasms. As critical regulators of growth, neurodevelopment, reproductive function, and energy homeostasis, thyroid hormones play fundamental roles in multiple physiological processes. Untreated hypothyroidism and hyperthyroidism are major global health issues with severe clinical effects (Taylor et al., 2018). The disease burden is further compounded by thyroid malignancies, with approximately 44,000 new cases of thyroid cancer diagnosed annually in the United States alone (Boucai et al., 2024). Recent progress in biomaterial science has opened novel therapeutic avenues, offering innovative approaches for the management of various thyroid disorders—among which hydrogels have emerged as a particularly promising class of materials due to their unique structural and functional characteristics. Hydrogels are three-dimensional (3D) network materials formed by hydrophilic polymers crosslinked physically or chemically, capable of absorbing large amounts of water without dissolving. Due to their structural similarity to the extracellular matrix (ECM), they possess good biocompatibility and tunable mechanical properties, leading to widespread application in the biomedical field. In thyroid disease treatment, hydrogels can serve as drug carriers for targeted delivery, scaffold materials for thyroid tissue engineering (Bernal-Chávez et al., 2022), and also for minimally invasive therapy. This article aims to review the latest research progress on hydrogels in thyroid disease diagnosis and treatment, analyze their advantages and challenges, and discuss future development directions. Figure 1 depicts the annual publication volume of studies related to hydrogels applied in the thyroid domain.

## 2 Basic characteristics and advantages of hydrogels

Hydrogels are 3D polymeric networks with swelling/collapse properties, flexibility, softness, biodegradability, and biocompatibility—traits well-matched to the thyroid gland, making them highly appealing for thyroid disease management. Their high water-absorbing capacity enables efficient loading of thyroid hormones (e.g., levothyroxine) and chemotherapeutics for targeted delivery (Bustamante-Torres et al., 2021), while their soft, porous structure mimics the thyroid extracellular matrix (TEM) to minimize mechanical irritation and immune responses that disrupt hormone secretion (Ho et al., 2022). Their structure can also be modified by varying monomer concentration, structure and functionality, as well as the cross-linker used (Chirani et al., 2015). Based on their origin, hydrogels are classified as natural, synthetic, or semi-synthetic hydrogels. Natural hydrogels possess inherent biocompatibility, bioactivity, and biodegradability but exhibit relatively poor stability and mechanical strength. Along with the responsive extent of physical properties, hydrogels are divided into conventional hydrogels and smart hydrogels based on their properties (Ho et al., 2022). Conventional hydrogels have only a small alteration consisting of swelling with external environmental conditions and low mechanical strength, whereas smart hydrogels are sensitive to the small changes in external environmental conditions, and immediately adjust their physical properties. Smart hydrogels include thermosensitive, pH-sensitive, and photo-sensitive types and so on (Ho et al., 2022). Over the past 60 years, hydrogels have been engineered to be implantable, injectable, and sprayable for many organs and tissues (Kopeček, 2009; Kouchak, 2014).

## 3 Application in the diagnosis of thyroid disease

Early and accurate diagnosis of thyroid disease—from benign/malignant nodule differentiation to occult metastasis detection—is clinically critical, as misdiagnosis or delayed detection causes improper treatment and poor prognosis. While conventional techniques like ultrasonography and magnetic resonance imaging (MRI) are widely used, they have inherent limitations, and hydrogels have emerged as promising candidates to address these issues, enhancing diagnostic accuracy and reliability (Chen et al., 2021; Hu et al., 2025).

Couplants are essential for ultrasonography, as conformal contact with skin and ultrasound probe is critical to ensure reliable imaging quality. Conventional liquid couplants cannot bear external compressive force, while solid couplants fail to fit curved skin surfaces well—this defect is prominent in cervical thyroid ultrasonography due to the anatomical curvature of the neck. A bilayer hydrogel acoustic couplant has been engineered for ultrasonography to adapt to the cervical curvature: a stiff upper layer resists probe pressure, and a compliant lower layer conforms closely to cervical curved skin, relieving stress concentration. This design overcomes the drawbacks of traditional media, enables high-quality thyroid nodule imaging, and establishes hydrogels as excellent alternatives for clinical thyroid ultrasonic imaging (Chen et al., 2021).

For molecular imaging, a deoxyribonucleic acid (DNA)-manganese (Mn) hydrogel (M-TDH) that selectively targets thyroglobulin (Tg) has demonstrated exceptional potential in MRIof occult metastatic and recurrent thyroid carcinoma. Its innovative principle lies in precise DNA sequence engineering—integrating Tg-specific single-strand DNA (ssDNA) aptamers that match the antigenic epitopes of Tg for high-affinity recognition, avoiding nonspecific binding. M-TDH has excellent biocompatibility, supported by normal liver/kidney function, no remarkable histological changes in major organs, effective *in vivo* clearance, and no abnormal Mn accumulation. This design specifically solves key clinical pain points such as poor specificity of traditional contrast agents, off-target effects of therapeutics, and difficulty in detecting occult metastases, enabling precise MRI of Tg-positive tumors (Hu et al., 2025).

Beyond imaging, hydrogels have also optimized diagnostic workflows. Although fine-needle aspiration biopsy (FNAB) remains the gold standard for thyroid-nodule evaluation (Uludag et al., 2023), skill acquisition is challenging. To address this, a hydrogel-based thyroid phantom—exhibiting high water retention and rapid self-healing—has been developed. The platform enables repeated, realistic FNAB practice, thereby accelerating and refining clinician training (Ma et al., 2024).

Additionally, hydrogel microparticles have been widely exploited in medical diagnostics, and crucially, shape modulation enables a sixfold reduction in the detection limit of thyroid-stimulating hormone (TSH) assays. As reported by Shapiro S.J. et al., this performance enhancement arises from an elevated bioassay signal, which is achieved by increasing the ratio of the hydrogel particle’s surface area to its 2-D projected imaging area for analysis (Shapiro et al., 2018).

## 4 Application in the treatment of thyroid disease

### 4.1 Non-surgical treatments

The thyroid gland is located in a neck region with rich neural and vascular networks, which imposes three strict design criteria on hydrogels intended for thyroid therapy: first, the ability to gel rapidly *in situ* and conform precisely to the thyroid’s anatomical structure; second, ultra-low immunogenicity to avoid adverse immune responses; third, on-demand payload release activated by local cues such as pH changes, enzymatic activity, or mild photothermal stimuli (Tang et al., 2025). These tailored properties make hydrogels ideal for non-surgical management of common thyroid disease.

#### 4.1.1 Hypothyroidism, hyperthyroidism, and thyroid eye disease

**FIGURE 2:**
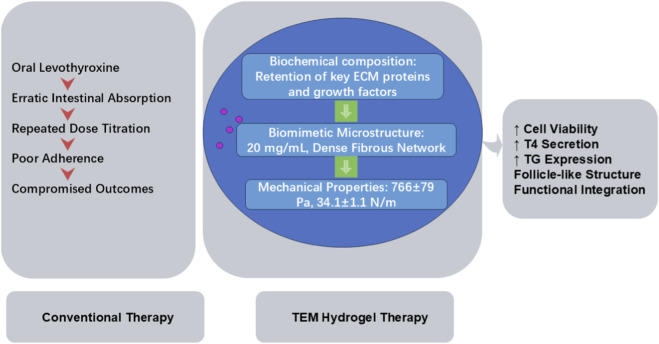
Mechanism and Advantage Comparison: Conventional Levothyroxine Therapy vs. TEM Hydrogel-based Autologous Thyroid Cell Construct for Hypothyroidism. ECM, thyroid extracellular matrix; TEM, thyroid extracellular matrix; T4, tetraiodothyronine; Tg, thyroglobulin.

Standard therapy for hypothyroidism remains lifelong oral levothyroxine replacement, yet erratic intestinal absorption and the need for repeated dose titration compromise patient adherence and treatment outcomes. A breakthrough 3D injectable hydrogel fabricated from decellularized TEM, with biosafety guaranteed by effective decellularization and low immunogenicity, closely replicates the native gland’s viscoelasticity and ultrastructure. When seeded with autologous thyroid cells, this TEM hydrogel-supported construct demonstrates superior viability, thyroid hormone secretion, and thyroid-related gene expression of the seeded cells, offering a promising sustained-release alternative to daily oral medications. Among the tunable material parameters of TEM hydrogels, three are most critical for promoting functional integration with thyroid tissue at both cellular and organ levels (supplemented with detailed data for partial hydrogel formulations): 1) Biochemical composition: Retention of key ECM proteins (collagens I/IV, fibronectin, laminin) and growth factors (vascular endothelial growth factor (VEGF), transforming growth factor-beta (TGF-β), fibroblast growth factor (FGF)) provides biochemical signals for cell adhesion and function; 2) Biomimetic microstructure: The dense, ordered fibrous network with suitable pore size (20 mg/mL TEM hydrogel) facilitates nutrient diffusion and cell organization; 3) Matched mechanical properties: Elastic modulus (766 ± 79 Pa) and toughness (34.1 ± 1.1 N/m) close to native thyroid tissue support cell morphology and tissue structural stability. These parameters synergistically enhance thyroid cell viability, tetraiodothyronine (T4) secretion, Tg gene/protein expression, and follicle-like structure formation, promoting functional integration (Zhang et al., 2024). The synergistic effects of biochemical, microstructural and mechanical properties of TEM hydrogel on thyroid cell function, as well as its superiority over conventional levothyroxine therapy, are illustrated in Figure 2.

**FIGURE 3:**
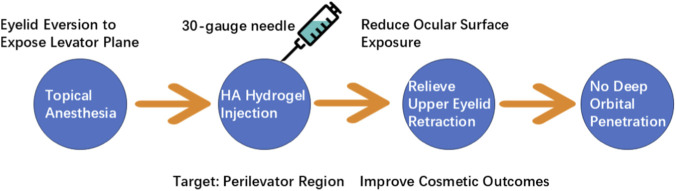
Clinical Application of HA Hydrogel in Graves’ Ophthalmopathy: Operational Steps and Safety Profile. HA, Hyaluronic acid.

Antithyroid drugs (e.g., methimazole, propylthiouracil) are limited by short half-lives, frequent dosing, and dose-dependent adverse effects; radioiodine or surgery carries additional risks (Wang et al., 2023). Hydrogel-based delivery systems can encapsulate these agents, providing controlled, localized release that extends dosing intervals, stabilizes plasma levels, and minimizes systemic toxicity. Hyaluronic acid (HA) hydrogel injection into the levator plane has recently been demonstrated to alleviate upper eyelid retraction, thereby improving both cosmetic outcomes and ocular surface exposure in patients with Graves’ ophthalmopathy (GO). This minimally invasive technique — performed under topical anesthesia with eyelid eversion to expose the levator plane and injection via a 30-gauge fine needle — avoids penetration into deep orbital structures and superficial ocular surfaces, confining HA delivery specifically to the perilevator region (Kohn et al., 2014). A stepwise minimally invasive procedure for HA hydrogel injection into the levator plane for Graves’ ophthalmopathy is depicted in Figure 3.

#### 4.1.2 Thyroid cancer management

Systemic drug delivery systems used in thyroid cancer treatment frequently face significant adverse effects, including nausea, loss of appetite, and hair loss, due to their impact on non-cancerous tissues (Yoo et al., 2018). Hydrogels offer innovative solutions in targeted drug delivery.

Accordingly, a local drug delivery system based on visible light-cured glycol chitosan (GC) hydrogel and doxorubicin (DOX) hydrochloride (GC10/DOX) was evaluated for thyroid cancer treatment (Yoo et al., 2018). A HA hydrogel loaded with Quercetin was tested alone and in combination with an inhibitor of Aurora Kinase type A and B (SNS-314), and it possesses anti-tumor activity in medullary and papillary thyroid cancer (Quagliariello et al., 2016). Cu(II)-based hydrogels loaded with paclitaxel were fabricated for thyroid cancer therapy, and their novel derivatives have been further optimized (Wang et al., 2024). An *in situ* spontaneously forming micelle-hydrogel system (iMHS) with programmable-release characteristics was developed for sequential chemotherapy for anaplastic thyroid carcinoma to reduce harsh side-effects (Yang et al., 2022).

To comprehensively evaluate the advantages, limitations, and clinical application potential of various hydrogel drug delivery systems for thyroid-related disorders, a horizontal comparative analysis was conducted based on core performance, applicable scenarios, and translational value, with the results summarized in Table 1.

**TABLE 1:** Comprehensive horizontal comparative analysis of hydrogel drug delivery systems for thyroid-related disorders.

Comparative dimensions	TEM hydrogel	HA gel (for thyroid eye disease eyelid retraction)	GC10/DOX hydrogel (for thyroid cancer)	HA-quercetin hydrogel (for thyroid cancer)	Cu(II)-CP/HA/CMCS@Paclitaxel hydrogel (for thyroid cancer)	iMHS (for anaplastic thyroid cancer)
Core advantages	1. Biomimetic 3D structure mimicking natural thyroid tissue; 2. Retains growth factors and ECM proteins; 3. Enhances thyroid cell viability, T4 secretion, and TG expression; 4. Forms follicle-like functional structures *in vitro*	1. Minimally invasive, repeatable, and reversible injection; 2. Effective for both active and inactive TED stages, bridging to surgery; 3. Few complications (only mild ecchymosis/edema)	1. Visible light-cured (10s irradiation) for on-demand gelation; 2. Dual-release pattern (initial burst + sustained release for 7 days); 3. Low systemic toxicity (avoids DOX cardiotoxicity); 4. *In vivo* validated antitumor efficacy (FTC-133 xenografts); 5. Adjustable crosslinking density adapts to variable tumor stiffness	1. CD44-mediated targeted delivery to tumor cells; 2. Synergistic cytotoxicity with SNS-314 (reduces IC50); 3. Dual antioxidant and anti-inflammatory activities; 4. Biocompatible matrix adapts to moderate tumor stiffness	1. Cu(II) CP enhances drug stability and cytotoxicity; 2. Modulates thyroid cancer biomarkers enables controlled paclitaxel release; 3. Reinforcement learning-optimized derivatives unvalidated clinically	1. Programmable sequential release of hydrophilic (DDP) and hydrophobic (PTX) drugs; 2. Long local retention with low systemic toxicity; 3. Prevents postoperative recurrence
Main disadvantages	1. Decellularization causes ∼50% GAG loss; 2. High concentration (30 mg/mL) impairs nutrient diffusion; 3. No *in vivo* therapeutic validation; 4. Limited to hypothyroidism	1. Transient effect (average 15 months, requires reinjection every 1–2 years); 2. Limited to mild eyelid retraction; 3. Uneven distribution under ultrasound; 4. No design optimization for TME stiffness/pressure	1. Requires visible light irradiation for gelation (may limit deep tumor application); 2. Initial burst release may cause local tissue irritation; 3. Only validated for FTC	1. Higher internalization efficiency in PTC than MTC; 2. Ineffective for low CD44-expression tumors; 3. Lack of long-term *in vivo* safety data	1. Cu(II) CP may induce potential metal ion toxicity *in vivo*; 2. No *in vivo* antitumor efficacy data; 3. Dependence on CP stability under physiological conditions; 4. No TME stiffness/pressure adaptation design	1. Complex preparation (precise micelle-hydrogel ratio control); 2. No effect on distant metastasis; 3. Human tolerance dose unconfirmed
TME adaptability (stiffness/pressure)	N/A- targets hypothyroidism (non-tumor microenvironment); mechanical properties match normal thyroid tissue	N/A	High - tunable crosslinking density adapts to variable stiffness	Moderate - biocompatible matrix adapts to moderate stiffness but not tunable; no targeted TIP reduction design	Low - No TME-specific optimization	High - porous structure reduces TIP
Clinical translation potential	High - provides alternative to hormone replacement for hypothyroidism, biomimetic advantages unique	High - addresses unmet transitional needs in TED, simple operation, low risk	High - In vivo safety and efficacy validated; TME adaptability (stiffness/pressure) improves translational potential	Medium - targeted synergy aligns with precision medicine, but needs TME-related optimization and larger clinical samples	Low - In vitro biomarker modulation promising, but lacks *in vivo* data and TME optimization	High - solves ATC chemotherapy limitations; TME adaptability enhances clinical applicability
Applicable scenarios	Thyroid tissue engineering for hypothyroidism	Mild upper eyelid retraction in TED (active/inactive), pre-surgical transition or palliative care	Local treatment of FTC; suitable for superficial tumors with variable stiffness accessible to light irradiation	Local treatment of medullary/papillary thyroid carcinoma	*In vitro* thyroid cancer cell inhibition; potential for local treatment of differentiated thyroid carcinoma	Local chemotherapy and postoperative residual tumor control in anaplastic thyroid carcinoma
Key distinctive features	Tissue-engineered scaffold for thyroid regeneration (hypothyroidism-focused)	Focuses on symptomatic relief of TED (cosmetic and functional improvement)	Light-triggered gelation enables precise local drug delivery	Emphasizes synergistic targeted therapy for differentiated thyroid cancer	Combines metal CP with hydrogel for dual-action (CP cytotoxicity + paclitaxel chemotherapy) and biomarker modulation	Specialized in sequential chemotherapy for aggressive ATC with TME adaptability

TME, tumor microenvironment; 3D, three dimensional; ECM, extracellular matrix; TG, thyroglobulin; GAG, glycosaminoglycan; N/A, not applicable; HA, hyaluronic acid; TED, thyroid eye disease; GC, glycol chitosan; DOX, doxorubicin; FTC, follicular thyroid carcinoma; IC50, half maximal inhibitory concentration; PTC, papillary thyroid cancer; MTC, medullary thyroid cancer; TIP, tissue interstitial pressure; CMCS, carboxymethyl chitosan; CP, coordination polymer; iMHS, *in situ* spontaneously forming micelle-hydrogel system; ATC, anaplastic thyroid cancer.

### 4.2 Surgical treatments

In medical applications, hydrogels can serve as ideal dressings for hemostasis and wound healing with advanced functions, promote the repair of thyroid cartilage and nerve injuries, and intelligent hydrogels hold potential for treating postoperative hypoparathyroidism, a common complication of total thyroidectomy.

#### 4.2.1 Hemostasis and wound healing

Winter’s seminal 1962 study established a cornerstone of modern wound care: an optimally moist microenvironment accelerates re-epithelialization and reduces scar formation (da Silva et al., 2019).

Hydrogels, 3D hydrophilic polymer networks, are uniquely equipped to deliver and maintain this moisture balance. Their inherent properties, including a unique structural design and high water content, grant them key advantages for wound care: excellent oxygen permeability, efficient absorption of wound exudate, and the ability to sustain a moist interface at the wound site. Beyond these basic functions, their tunable physical and chemical characteristics allow them to mimic the composition and mechanical properties of natural tissues, thereby providing sufficient space and mechanical support for cell migration and tissue regeneration (Liang et al., 2021).

In addition to these fundamental roles, hydrogel dressings exhibit a range of advanced functionalities, as reviewed in existing literature (Arabpour et al., 2024). These include antimicrobial activity, adhesion and hemostatic effects, anti-inflammatory and antioxidant capabilities, substance delivery, self-healing, stimulus responsiveness, conductivity, and the recently developed wound monitoring feature. Among these, the anti-inflammatory function of hydrogel dressings has seen significant advancements in recent years, which has helped address multiple clinical challenges encountered in efforts to promote wound healing (Arabpour et al., 2024). Notably, hydrogels integrated with monitoring functions further contribute to optimized care by enabling continuous evaluation of the wound microenvironment, with the generated data serving as crucial guidance for formulating treatment strategies (Liang et al., 2021).

Nevertheless, physically cross-linked hydrogels are inherently limited by relatively low mechanical properties and poor structural stability, which severely restricts their clinical application in dynamically active wound sites (e.g., the neck or joints with frequent movement), but such limitations can be improved through various cross-linking methods. In contrast, chemically cross-linked hydrogels are known for their strong mechanical attributes and excellent stability. Remarkably, Wu et al. have developed a chitosan/alginate hydrogel dressing incorporated with bioglass (BG) as a multifunctional bioactive ingredient, which enhances mechanical stability and bioactivity (Arabpour et al., 2024; Wu and Li, 2021).

#### 4.2.2 Repair of thyroid cartilage and laryngeal nerve injuries

Peripheral nerve injuries and thyroid cartilage defects both present significant clinical challenges due to their limited self-healing capabilities. For injured peripheral nerves, end-to-end anastomosis is the first-choice treatment; however, when faced with large nerve defects, autologous transplantation becomes the most common approach, which unfortunately carries the risk of inducing additional neural disorders (e.g., neuroma) at the donor sites. Similarly, thyroid cartilage, characterized by its avascular nature, exhibits extremely limited self-repair capacity. Current clinical strategies for repairing thyroid cartilage defects share similarities with approaches used for peripheral nerve repair, including autologous or allogeneic cartilage transplantation, end-to-end anastomosis, and the application of metal-based scaffolds (Li et al., 2025; Yoshimatsu et al., 2020).

To address the limitations of these thyroid cartilage repair approaches, various hydrogel-based bioinks have been developed for 3D-printed cartilage scaffolds: sodium alginate, gelatin, tannic acid, and calcium chloride were cross-linked to fabricate a hydrogel-based bioink, and the resulting hydrogel-derived scaffolds effectively promoted cartilage regeneration in rabbits within 8 weeks, with only a small amount of scaffold residue remaining *in vivo* at 12 weeks; these scaffolds also exhibited non-toxic degradation products and excellent biocompatibility (Li et al., 2025). Among existing advanced materials, Pluronic F127 diacrylate (F127DA) nano-micelle crosslinked methacrylated hyaluronic acid (MeHA) hydrogels (NMgels) with outstanding compressive properties have been verified to facilitate rabbit cartilage regeneration within 8 weeks. Specifically, the F15H1.5 NMgel—a nanomicelle-crosslinked hydrogel composed of 15 wt% F127DA micelles and 1.5 wt% MeHA—showed an *in vitro* degradation rate of approximately 34.2% over 10 days under enzymatic conditions (5 U mL^−1^ hyaluronidase, pH 7.4 phosphate-buffered saline, 37 °C) (Ren et al., 2019). Additionally, Arguchinskaya NV et al. successfully printed a thyroid cartilage scaffold using collagen hydrogel as the primary component (with proven excellent biocompatibility: rat chondrocytes cultured on the scaffold achieved a survival rate of 88.1% ± 5.3% on day 3 and further increased to 94.5% ± 5.2% on day 7) and gelatin hydrogel as a temporary support. The gelatin component can be easily removed at 37 °C without damaging the main scaffold, providing novel insights and practical experience for the clinical reconstruction of laryngeal cartilage defects (Arguchinskaya et al., 2021). However, collagen inherently exhibits weak mechanical properties and rapid biodegradation, which may compromise the long-term *in vivo* stability of the scaffold. Meanwhile, biocompatible composite hydrogels incorporating magnetic nanoparticles have also demonstrated promising application potential in cartilage tissue engineering (Piantanida et al., 2019). In the realm of nerve repair, RADA16-I hydrogel, with properties similar to ECM, is effective for recurrent laryngeal nerve (RLN) regeneration and thyroarytenoid muscle atrophy prevention in a rat model of RLN injury, as confirmed by laryngoscopy, electrophysiological and histological examinations at 8 weeks post-injury (Yoshimatsu et al., 2020).

Similarly, chitosan-acrylic acid-acrylamide-dopamine acrylamide semi-interpenetrating polymer network (semi-IPN) hydrogels loaded with tacrolimus (FK506) locally can enhance macrophage recruitment, induce the polarization of macrophages toward the M2 phenotype, and establish a neuroprotective microenvironment. Consequently, by virtue of the synergy between immunomodulation and biomechanical adaptation, these hydrogels further facilitate myelin sheath reconstruction to support peripheral nerve regeneration. Local administration of FK506 is safer and more effective in treating peripheral nerve injury than systemic delivery, as it avoids the associated systemic immunosuppression and subsequent refractory infections. Twenty-eight days after hydrogel implantation in rats, hematoxylin-eosin (HE) staining of major organs (heart, liver, spleen, lung, kidney) showed no acute or chronic pathological toxicity and no significant histological abnormalities, confirming the good *in vivo* biosafety of the material (Wang et al., 2022).

#### 4.2.3 Prevention and management of postoperative hypoparathyroidism

Hypoparathyroidism is a prevalent postoperative complication of total thyroidectomy that severely impairs patients’ quality of life, representing a critical and urgent unmet clinical need following thyroid surgery. Calcium and vitamin D supplementation can secondarily induce progressive renal calcium deposition, which ultimately culminates in renal insufficiency. Additionally, exogenous parathyroid hormone (PTH) injections often trigger marked fluctuations in serum calcium levels, while the survival efficacy of autologous parathyroid transplantation remains unsatisfactorily low and difficult to guarantee. Thus, there is an imperative need to explore novel therapeutic strategies for hypoparathyroidism. Notably, Gokyurek M et al. proposed an intelligent porous hydrogel platform with great potential for culturing parathyroid organoids (Gokyurek et al., 2023). This platform effectively enhances parathyroid organoids’ long-term survival and hormone responsiveness, and supports functional thyroid cell culture, making it a potential treatment for both hypoparathyroidism and hypothyroidism. Specifically, its biomimetic structure maintains parathyroid cells’ globular morphology and 14-day viability, preserves surface calcium receptors (CaSR) expression, and enables physiological Ca^2+^-dependent PTH secretion (low Ca^2+^ promotes, high Ca^2+^ inhibits). Furthermore, PTH aids fracture healing and implant fixation by accelerating skeletal repair. Thus, future research directions for this hydrogel include coculturing parathyroid and bone cells to explore their interactions (Gokyurek et al., 2023).

## 5 Challenges and prospects

Although hydrogels show great potential in thyroid disease treatment, they still face several key challenges. First, the thyroid’s special anatomical location complicates precise drug delivery, while the biocompatibility and degradability of long-term implantable hydrogels require further optimization. Second, clinical translation is hindered by insufficient large-scale preclinical safety data, unclear subtype-specific dosing schedules, and a lack of standardized manufacturing processes. Third, regulatory and economic barriers persist due to unestablished biosafety requirements and industry standards for hydrogel-based thyroid therapies. Notably, a critical yet understudied knowledge gap—urgently awaiting resolution—lies in the adaptability of hydrogel-supported thyroid metabolic systems to physiological endocrine feedback: i.e., whether hydrogel-seeded thyroid cells have yet to demonstrate responsiveness to hypothalamic-pituitary-thyroid (HPT) axis signals and the ability to dynamically adjust hormone secretion via feedback loops. This functional regulatory gap directly affects engineered thyroid constructs’ capacity to mimic the natural thyroid’s homeostatic control, a key prerequisite for successful clinical translation.

Nevertheless, promising evidence from the literature reviewed herein confirms hydrogels inherently possess the potential to address many of these limitations. For instance, hydrogels exhibit tunable degradability, rarely elicit immune responses from long-term implantation, and their degradation products show no adverse effects *in vitro* and *in vivo* (Hu et al., 2025; Li et al., 2025; Arguchinskaya et al., 2021; Piantanida et al., 2019). Moreover, hydrogels can safely support gene editing and bioartificial thyroid construction via low-immunogenic, biodegradable matrices with favorable autologous cell compatibility (Zhang et al., 2024)—effectively avoiding immune rejection and toxic side effects—while their biomimetic 3D microenvironment preserves edited cell viability, acts as a protective carrier for gene editing tools, and enables multicellular assembly and nutrient exchange to form functional constructs.

This synergistic combination of hydrogels and gene-based therapeutic strategies has been well validated in wound healing applications, and gene therapy is undoubtedly an emerging technology for wound healing. It leverages nucleic acid molecules to regulate cell functions at the genetic level, thereby promoting wound repair, and boasts prominent advantages: high target selectivity derived from sequence-specific recognition, prolonged duration of action due to sustained genetic regulation, enhanced action specificity that minimizes off-target effects, and flexible sequence designability to adapt to diverse wound healing needs. However, gene therapy for wound repair still faces notable challenges across key dimensions: (1) targeting limitations: poor cellular penetration hindered by molecular size and other specific characteristics; (2) safety concerns: inherent immunogenicity as exogenous substances, potential off-target risks, and hepatic burden from drug accumulation with repeated administration; (3) operational complexity: reliance on advanced delivery systems to overcome stability and penetration barriers, increasing development and application difficulty; (4) clinical translation hurdles: need for standardized protocols for dosage control and long-term safety monitoring, slowing the transition from preclinical research to clinical application. To address these limitations, a tripartite integration of nucleic acid therapy, nanocarriers and hydrogel dressings has been proposed as an effective solution, which enables enhanced nucleic acid delivery efficiency and cellular uptake, as well as localized and sustained release of nucleic acid drugs for durable therapeutic effects on wounds (Zhang et al., 2022).

Notably, this interdisciplinary integration logic—using hydrogels to overcome the limitations of traditional delivery systems (e.g., poor targeting, systemic toxicity)—is equally applicable to thyroid cancer therapy, where hydrogels exhibit distinctive values particularly prominent when compared to conventional nano-platforms (e.g., nanoparticles, nanoliposomes). Unlike nano-carriers that rely on systemic circulation for delivery, are prone to rapid clearance, and suffer from off-target distribution, hydrogels have achieved three core breakthroughs in thyroid cancer treatment by virtue of their high biocompatibility, tunable mechanical properties, and stimulus-responsive characteristics. Firstly, through *in situ* gelation and localized sustained-release mechanisms, hydrogels can precisely anchor chemotherapeutic agents, tyrosine kinase inhibitors (TKIs), or ^131^I at the tumor site (Tang et al., 2025). While enhancing local drug concentrations, they avoid the systemic toxicity induced by traditional nano-platforms, which is especially compatible with the anatomical feature of dense neurovascular networks surrounding the thyroid gland in the neck. Secondly, hydrogels break through the limitation of the single delivery function of nano-carriers—by loading cytokines, immune checkpoint inhibitors, or vaccine adjuvants, they can remodel the immunosuppressive tumor microenvironment, transforming “cold” lesions such as poorly differentiated thyroid carcinoma (PDTC) into “hot” immune-responsive sites. This immunomodulatory capacity is a unique advantage that traditional nano-platforms lack, providing new possibilities for improving the efficacy of immunotherapy in thyroid cancer. Thirdly, hydrogels mimic the extracellular matrix (ECM) structure, enabling them to not only serve as drug delivery vehicles but also function as scaffolds for post-resection tissue repair, preventing cervical adhesions and supporting the bioprinting of thyroid organoids (Tang et al., 2025). This multifunctionality addresses the unmet clinical needs of comprehensive management for thyroid cancer patients, going far beyond the single therapeutic role of conventional nano-platforms. Collectively, these unique properties make hydrogels a promising candidate to redefine locoregional control in thyroid cancer therapy, with great potential to mitigate systemic toxicity and improve patients' survival and quality of life.

Building on the validated hydrogel-based delivery strategies (from wound healing) and the proven advantages of hydrogels in thyroid cancer therapy, hydrogels for thyroid disease treatment have clear and promising future development directions to overcome existing obstacles and accelerate clinical translation. These research priorities focus on multiple interdisciplinary fronts, including developing multi-responsive smart hydrogel systems to achieve more precise disease treatment, combining advanced gene editing technology to construct functional bioartificial thyroids, integrating nanotechnology and imaging technology to realize integrated diagnosis and therapy (theranostics), and exploring 3D bioprinting technology to prepare personalized thyroid tissue constructs. With the in-depth convergence and development of materials science, molecular biology and clinical medicine, hydrogel technology is expected to bring revolutionary breakthroughs in the clinical treatment of thyroid diseases, and further provide safer and more effective novel therapeutic options for patients with thyroid disorders, especially those with advanced or recurrent thyroid cancer.
